# Identification of novel biomarkers and candidate small-molecule drugs in cutaneous melanoma by comprehensive gene microarrays analysis

**DOI:** 10.7150/jca.49702

**Published:** 2021-01-01

**Authors:** Jilei Ma, Xin Cai, Li Kang, Songfeng Chen, Hongjian Liu

**Affiliations:** 1 Department of Clinical Laboratory, The First Affiliated Hospital of Zhengzhou University, Zhengzhou, Henan, 450052, China.; 2 Department of Biotechnology and Food Engineering, Anyang Institute of Technology, Anyang, Henan 455000, PR China; 3 Department of Human Anatomy and Histoembryology, Henan Vocational College of Nursing, Anyang, Henan, 400500, China.; 4 Department of Orthopaedics, The First Affiliated Hospital of Zhengzhou University, Zhengzhou 450052, China.

**Keywords:** cutaneous melanoma, novel biomarkers, candidate small molecules, prognostic factor

## Abstract

**Background:** Melanoma is a pernicious skin cancer with high aggressiveness. This study aimed to identify potential novel biomarkers associated with the prognosis and pathogenesis of cutaneous melanoma and to explore new targeted drugs for melanoma. **Methods:** Two Gene Expression Omnibus (GEO) microarray datasets, GSE3189 and GSE7553 were combined to analyze the differentially expressed genes (DEGs). To better understand the DEGs in the melanoma pathogenesis, we performed gene enrichment analyses and established a protein-protein interaction network (PPI). The survival analyses for key genes were conducted based on the GEPIA platform. Finally, we mined the CMap database to explore potential small-molecule drugs to target the obtained DEGs. **Results:** In short, we identified 500 DEGs between cutaneous melanoma samples and normal samples. The PPI network was established with 349 nodes and 1251 edges. Signaling pathway analysis showed that these genes play a vital role in ECM-receptor interactions, the PPAR signaling pathway and pathways in cancer. Eight DEGs with a relatively high degree of connectivity (CDC45, CENPF, DTL, FANCI, GINS2, HJURP, TPX2 and TRIP13) were selected as hub-genes that remarkably correlated to a poor survival rate. Based on 500 DEGs, 20 small-molecule drugs that potentially target genes with abnormal expression in cutaneous melanoma were obtained from the CMap database. Among these compounds, we found that menadione has the greatest therapeutic value for melanoma. **Conclusions**: In conclusion, we identified the 8 candidate biomarkers and potential key signaling pathways in cutaneous melanoma through comprehensive microarray analyses. The identified candidate drugs have provided several directive significances for the synthesis medicine for melanoma.

## Introduction

Melanoma, which arises from melanocytes, usually occurs on the skin, with high aggressiveness and mortality. Up to 200,000 cases of malignant melanoma are registered each year based on WHO statistical data 1-4. Although the majority of patients diagnosed with local melanoma have a good outcome, prognosis remains poor for those with high-risk or advanced metastatic melanoma 5. Current therapeutic modalities include chemotherapy, immunotherapy, targeted therapy and surgical resection. In recent 10 years, a variety of therapeutic strategies have been developed for melanoma based on the identification of molecular factors associated with the pathogenesis and prognosis of melanoma.

Numerous genomic alterations in the PI3K and MAPK signal transduction pathways play vital roles in the molecular pathogenesis of melanoma. In addition, the microenvironment and immune system are significantly associated with the melanoma occurrence and development. For example, the BRAF V600 mutation in melanoma can result in the suppression of melanoma antigen and the reemergence of an immunosuppressive tumor microenvironment through constitutive activation of the MAPK pathway. Nowadays, some targeted therapeutic strategies, including BRAF and MEK inhibitors, have enhanced survival benefits for melanoma patients, but treatment failure caused by drug resistance remains an obstacle 6-10. Therefore, identifying the potential novel biomarkers and exploring new targeted drugs for melanoma can help solve this problem.

Herein, we demonstrated the differentially expressed genes (DEGs) determined by computational bioinformatics analysis based on the profiles of GSE3189 and GSE7553 from the GEO database. Meanwhile, our work explored some candidate small molecules that may reverse gene expression in melanoma by mining the CMap database. The results of the present research provide new promising biomarkers that could help identify new therapeutic targets for melanoma.

## Materials

### Gene Microarray Data

From the Gene Expression Omnibus (GEO) database, we downloaded and analyzed two gene microarray datasets, GSE3189 dataset and GSE7553 dataset. GSE3189 was based on platform GPL96 and included 52 samples, containing 45 tumor samples and 7 normal samples. GSE7553 was based on platform GPL570 and contained 58 samples, including 54 tumor and 4 normal samples. Their clinical properties of them are shown in Table 1.

### DGEs screening and enrichment analysis

The downloaded original CEL files were divided into melanoma group and normal control groups. The normalization of raw data was performed based on the affy package. We applied the Limma package to evaluate DEGs between two above groups 11. The identification criteria for DEGs screening were P value < 0.01 and |logFC| > 1. To further explore the association between the obtained DEGs and the pathogenesis of melanoma, we conducted gene enrichment analyses including Gene Ontology (GO) and KEGG pathway analyses. Enriched GO analysis was performed to investigate the core biological processes (BP), cellular components (CC) and molecular functions (MF) using DAVID 12. We performed KEGG enrichment analysis to elucidate the potential signaling pathways associated with overlapping DEGs. The statistically significance was defined as P< 0.05.

### PPI network establishment and module analysis

All identified DEGs were submitted to the STRING database to construct their protein interactions 13. An integrated value > 0.4 was deemed to be significant and then PPI networks were constructed according to the obtained values. Subsequently, significant modules were separated from the PPI network by Molecular Complex Detection (MCODE) 14. We also performed enrichment analyses for these significant modules. We visualised the core biological process (BP) of hub genes using the BiNGO plugin of Cytoscape 15.

### Survival analysis of hub genes

The UCSC Cancer Genomics Browser was used to construct hierarchical clustering of module genes. We then constructed a co-expression network of module genes via the cBioPortal platform 16. To further verify our results, we explored the ability of hub genes to predict patients' prognosis using the GEPIA database 17. We also calculated the hazard ratio (HR) of overall survival between high- and low- expression groups. The differences in the protein levels of hub genes between melanoma and normal tissues were evaluated using the human protein atlas database.

### Small-molecule drug identification

Small-molecule drugs with potential value for treating cutaneous melanoma were identified using the CMap platform, which stores a large number of gene expression profiles induced by various small molecules 18. We divided the gene expression of melanoma into high- and low-expression groups, and then submitted them to the CMap online platform. CMap eventually exhibits an enrichment score for each small molecule, and the closer the score is to -1, the greater its potential for treating melanoma.

## Results

### DEGs identification

**Figure 1:**
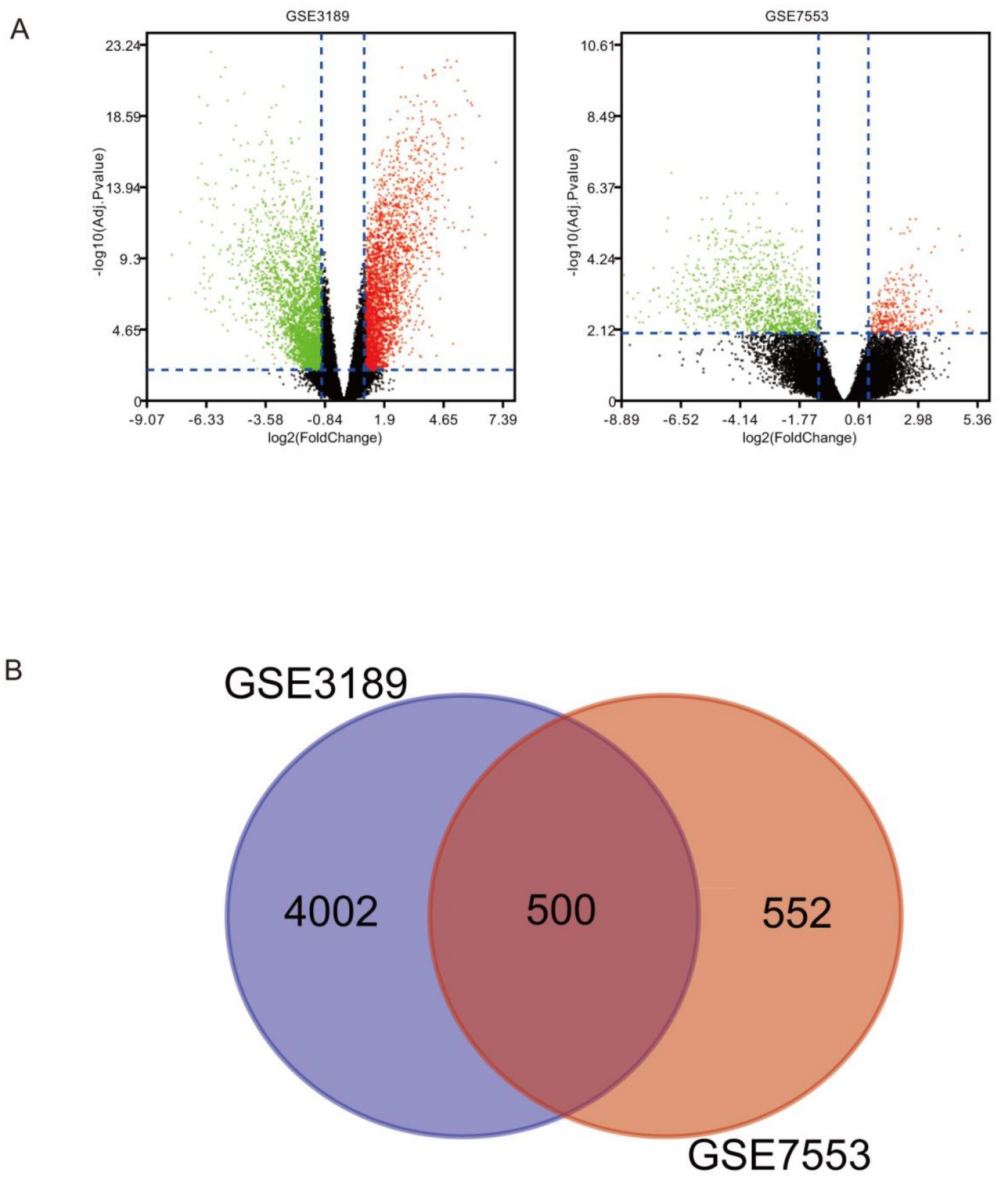
** (A)** DEGs between melanoma and normal tissues were identified via the Limma package, and visualized using a volcano plot. **(B)** Venn diagram revealing 500 overlapping DEGs from GSE3189 and GSE7553 datasets.

There were 500 overlapping DEGs identified via the Limma package in our study. Figure 1A shows s volcano plot of DEGs of melanoma. The Venn diagram shown in Figure 1B illustrates the 500 overlapping DEGs between the two datasets.

### Enrichment analyses

**Figure 2:**
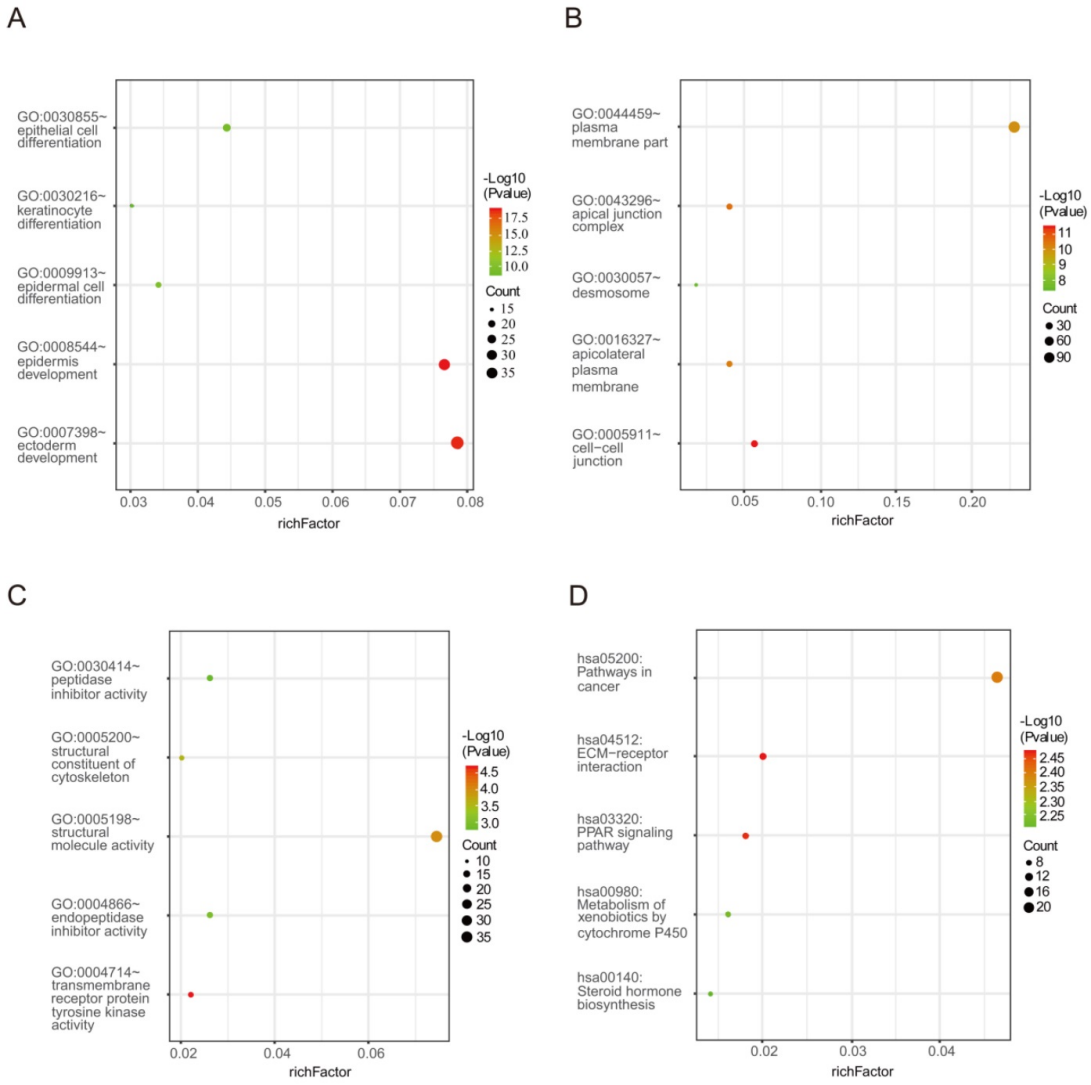
The enrichment analysis for DEGs including GO and KEGG pathway analyses. **(A)** Biological processes **(B)** Cellular components **(C)** Molecular functions **(D)** KEGG signaling pathway enrichment analyses.

Biological process analysis revealed that the obtained DEGs were mainly enriched in epidermal cell differentiation, epidermis development, ectoderm development, epithelial cell differentiation and keratinocyte differentiation. Analysis of cellular components manifested that these DEGs were particularly relevant to cell-cell junction, the apical junction complex, apicolateral plasma membrane, plasma membrane part and desmosome. Similarly, variations in the molecular functions of DEGs were significantly enriched in transmembrane receptor protein tyrosine kinase activity, structural molecular activity, structural composition of the cytoskeleton, endopeptidase inhibitor activity and peptidase inhibitor activity. In addition, Figure 2 and Table 2 reveal that DEGs were mainly involved in signaling pathways including ECM-receptor interaction, the PPAR signaling pathway, pathways in cancer, metabolism of xenobiotics by cytochrome P450 and steroid hormone biosynthesis.

### Construction of the PPI network and module analysis of DEGs

**Figure 3:**
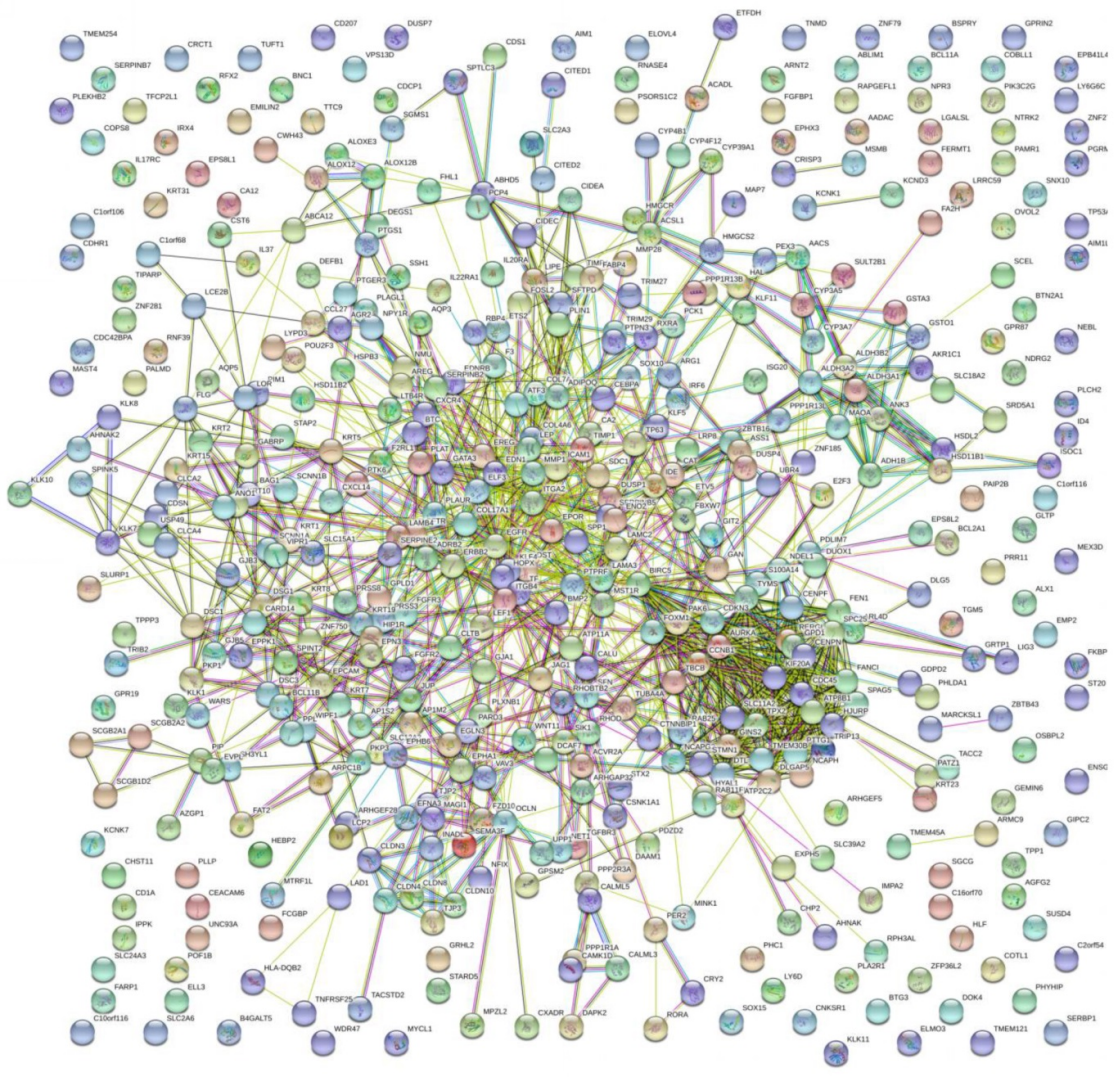
PPI network of DEGs with 349 nodes and 1251 edges was constructed according to the STRING database.

**Figure 4:**
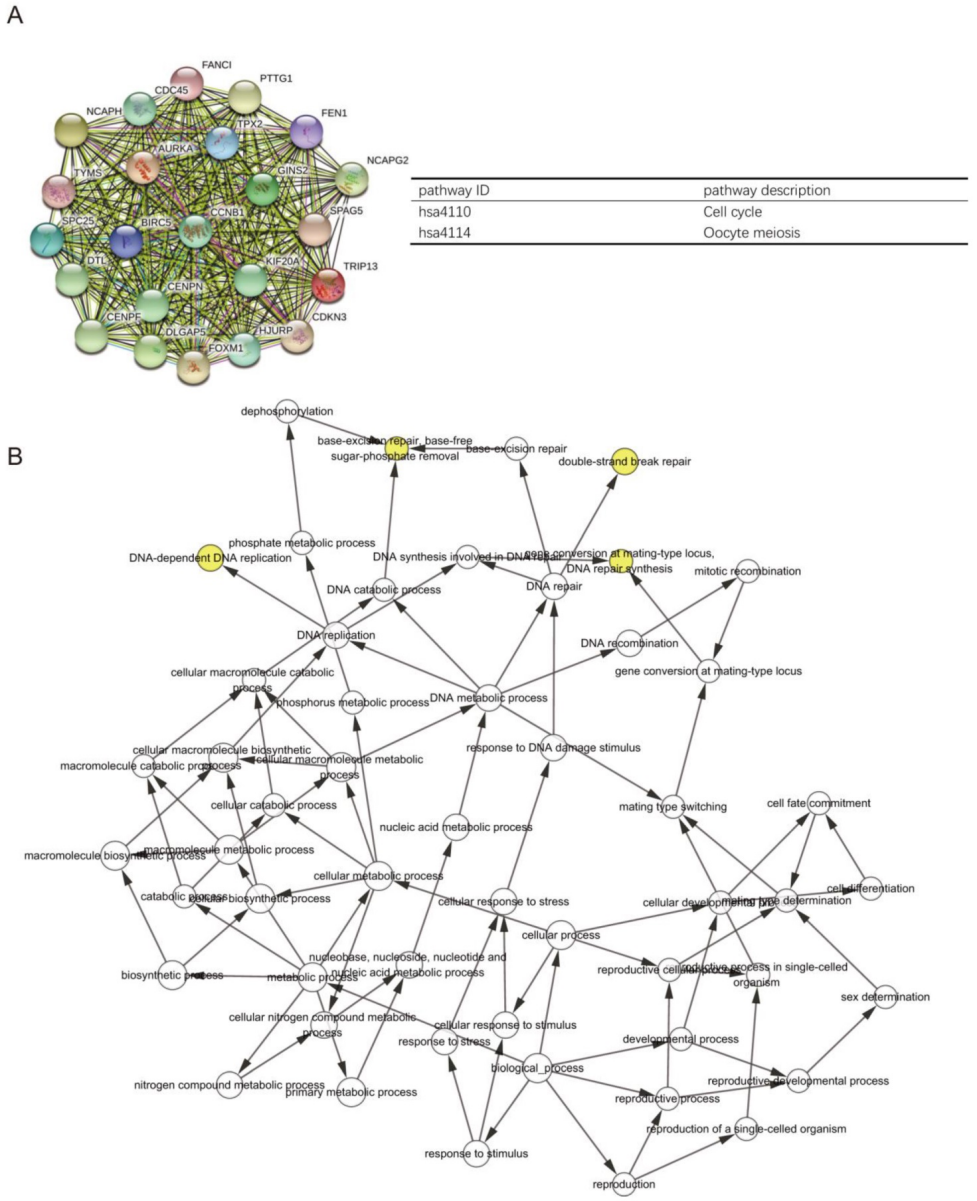
** (A)** PPI network construction revealed the most significant modules, and signaling pathway enrichment analyses were conducted for the module genes. **(B)** The BiNGO plugin of Cytoscape was used to explore the biological processes involved in module genes.

**Figure 5:**
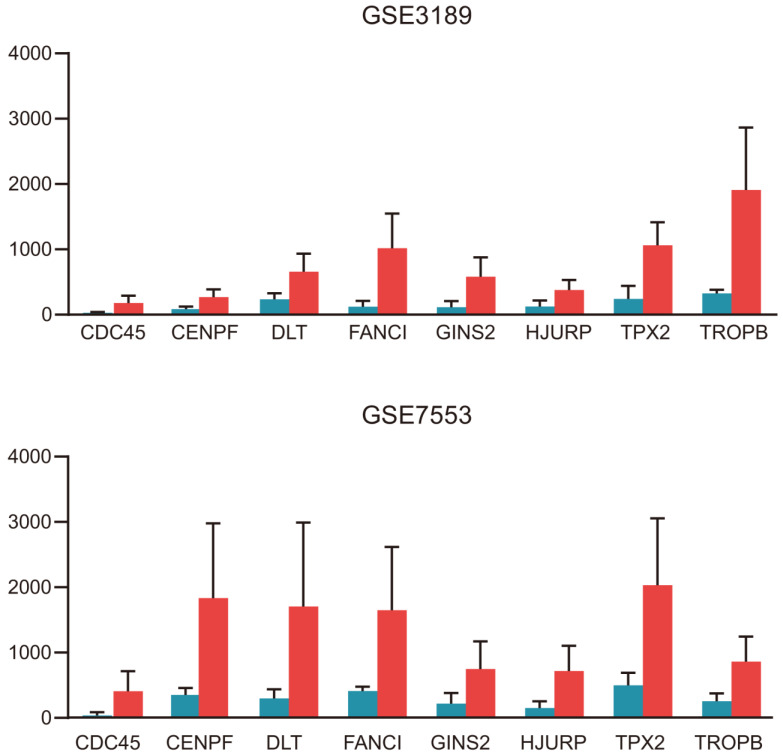
The significantly upregulated expression levels of CDC45, CENPF, DTL, FANCI, GINS2, HJURP, TPX2 and TRIP13 in melanoma tissues among each dataset.

The PPI network of DEGs with 349 nodes and 1251 edges was constructed according to the STRING database (Figure 3). MCODE was adopted to extract the module with the highest node degree by screening the above PPI network. The pathways of cell cycle and ocyte meiosis were markedly associated with the module genes (Figure 4A). GO analysis by BinGo demonstrated that the module genes were markedly correlated with DNA-dependent DNA replication, base-free sugar-phosphate removal, base-excision repair and double-strand break repair (Figure 4B). Eight hub genes with high connectivity, CDC45, CENPF, DTL, FANCI, GINS2, HJURP, TPX2 and TRIP13, whose expression in melanoma was significantly up-regulated than in normal tissues, were selected for deeper analyses (Figure 5).

### Prognostic value of hub genes

**Figure 6:**
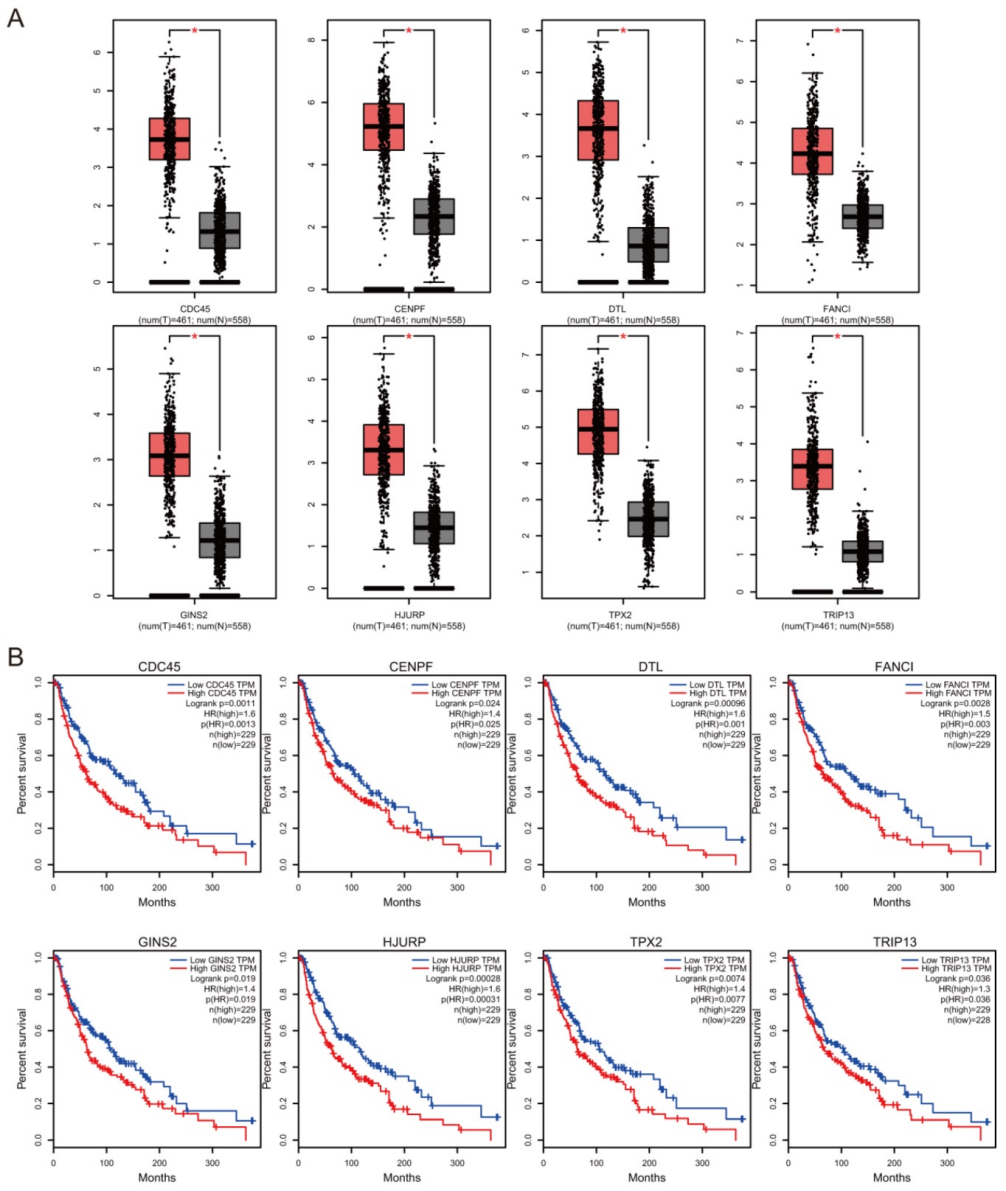
The hub gene expression and their prognostic value were validated using GEPIA database. High expression levels of CDC45, CENPF, DTL, FANCI, GINS2, HJURP, TPX2 and TRIP13 were significantly correlated with lower overall survival for melanoma patients.

**Figure 7:**
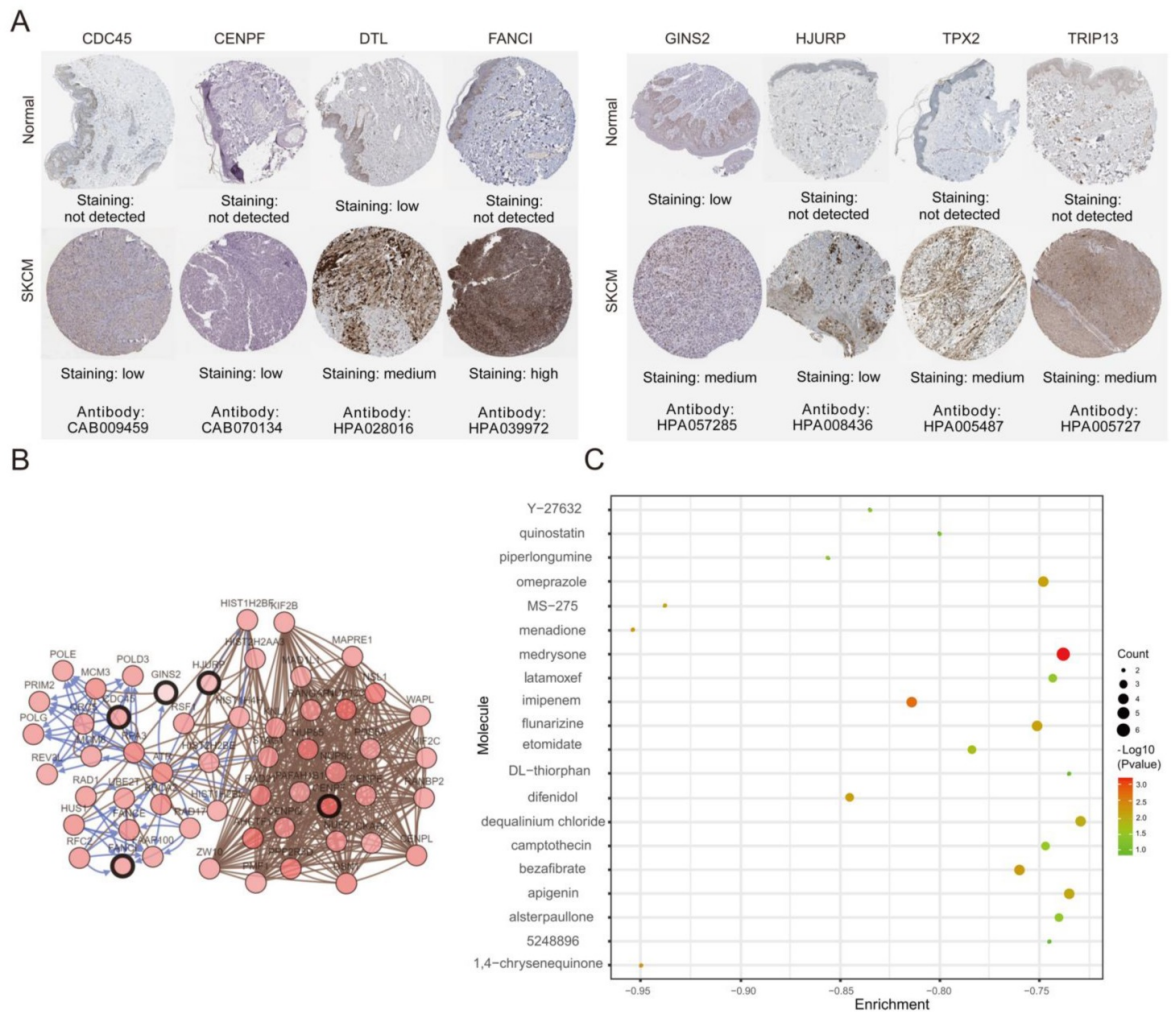
** (A)** The protein expression of hub genes was markedly upregulated in melanoma tissues compared to that in normal tissues. **(B)** A co-expression network of module genes was established via cBioPortal platform. Nodes with thick outlines correspond to hub genes; those with thin outlines indicate co-expression genes. **(C)** The CMap platform and the overlapping genes were utilized to predict small-molecule drugs with potential value in the treatment of cutaneous melanoma.

The association between hub genes expression and pathological features of melanoma was further confirmed by the HPA database and the cBioPortal. Based on GEPIA database analysis, the above hub genes exhibited obvious differences in expression between melanoma and control tissues. This difference validated again that these hub genes were highly expressed in melanoma tissues and closely associated with the onset of melanoma (Figure 6A). Overall survival (OS) analysis of total 458 melanoma patients was obtained from the GEPIA database, and the cancer patients were also divided into two groups according to median expression levels. It was found that upregulation of CDC45, CENPF, DTL, FANCI, GINS2, HJURP, TPX2 and TRIP13 was correlated with remarkably decreased OS in melanoma patients. (Figure 6B) The expression levels of CDC45, CENPF, DTL, FANCI, GINS2, HJURP, TPX2 and TRIP13 may be considered as crucial prognostic predictors of melanoma patients. HPA database analysis reconfirmed significantly higher expression levels of CDC45, CENPF, DTL, FANCI, GINS2, HJURP, TPX2 and TRIP13 in cancer tissues than in adjacent normal tissues at the protein level (Figure 7A). A co-expression network of the module genes was shown in Figure 7B.

### Potential small-molecule drugs identification

To identify potential small-molecule therapeutic candidates capable of reversing genetic changes in melanoma, we conducted the computational bioinformatics analysis of DEGs using the CMap database. An enrichment value closer to -1 indicated that the small molecules had a greater ability to reverse melanoma gene expression. The 20 most significant small molecules were identified, of which menadione and 1,4-chrysenequinone were the most likely to reverse the melanoma gene expression (Table 3 and Figure 7C). These candidate small-molecule drugs may ameliorate the tumor condition of melanoma; thus, they are potential new targeted drugs that may be explored for melanoma treatment. However, the role of the underlying molecular mechanisms of these candidate small molecules in melanoma should be clarified in the next work.

## Discussion

Recently, high-throughput sequencing technologies have been widely used to explore the key genetic or epigenetic changes in the malignant progression of melanoma. Moreover, integrated bioinformatics analysis has been widely used for screening novel biomarkers, hub node discovery from PPI networks and prognostic analysis 19. Our study performed comprehensive microarray analyses to identify novel biomarkers for cutaneous melanoma, and to explore small-molecule drugs according to the melanoma gene expression profiles, with the aim of providing new therapeutic strategies for cutaneous melanoma.

The 500 overlapping DEGs identified were significantly associated with the occurrence and progression of melanoma. It is suggestive that these overlapping DEGs may be novel diagnostic, prognostic or personalized therapeutic biomarkers. GO enrichment analysis for these overlapping DEGs revealed the potential molecular mechanisms underlying melanoma pathogenesis. Epidermal cell differentiation, epidermal development, and ectodermal development were the three most important functions identified among the biological processes. Molecular function analysis indicated the involvement of DEGs in transmembrane receptor protein tyrosine kinase activity, structural constituents of the cytoskeleton, and structural molecule activity. Variations in cell components were mainly related with the apical junction complex, cell-cell junction, and apicolateral plasma membrane. Additionally, the main biological pathways with enriched overlapping DEGs included ECM-receptor interaction, the PPAR signaling pathway, pathways in cancer, metabolism of xenobiotics by cytochrome P450 and steroid hormone biosynthesis. It was previously reported that Twist2 transcriptionally regulates the ECM-receptor interaction pathway and contributes to kidney cancer cell proliferation and invasion 20. The expression of β-catenin and its downstream effector, SOX9, can be suppressed by PPARγ 21. In summary, our bioinformatics analysis results were supported by these previous reports.

The PPI network among the overlapping DEGs revealed the most significant network module. CDC45, CENPF, DTL, FANCI, GINS2, HJURP, TPX2 and TRIP13 with high connectivity in the network were finally defined as hub genes in our study. To verify the results of bioinformatics analysis, hub gene expression levels and their ability to predict patients' prognosis were determined using the GEPIA database. Mining of the GEPIA database further confirmed the same expression trend found in the GEO database and CDC45, CENPF, DTL, FANCI, GINS2, HJURP, TPX2 and TRIP13 expression had a significant influence on the prognosis of melanoma patients. High expression of CDC45, CENPF, DTL, FANCI, GINS2, HJURP, TPX2 and TRIP13 were significantly correlated with worse overall survival in melanoma patients. The immunohistochemical analysis from HPA database demonstrated that the expression of these eight hub genes was consistent with their protein content, thereby verifying the accuracy of our findings. Our work is the first to reveal the diagnostic, prognostic and therapeutic value of these eight hub genes in melanoma, which could provide new insights regarding melanoma.

Whether these eight key genes play a part in the initiation and progression of melanoma has not been reported before. To further explore the potential mechanism of CDC45, CENPF, DTL, FANCI, GINS2, HJURP, TPX2 and TRIP13 in the pathogenesis of melanoma and enhance our understanding of this multi-gene hereditary disease, we predicted potential transcription factors that may regulate their expression and constructed a molecular regulatory network of lncRNA-miRNA-mRNA for these genes. This genetic regulatory network will contribute to elucidating the relationship between hub genes and melanoma initiation and progression. Jing et al. demonstrated that CDC45 is upregulated in papillary thyroid carcinoma and that depletion of CDC45 can suppress tumor growth 22. CENPF is closely associated with cell proliferation and is upregulated in multiple cancers, such as nasopharyngeal cancer, hepatocellular carcinoma, esophageal squamous cell carcinoma, gastrointestinal stromal tumors and breast cancer 23-25. FANCI can prevent the replication stress of carcinogenic DNA through its role in DNA repair, and regulate the activity of the Akt oncogene by promoting the inhibitory function of PHLPP 26. In mammals, HJURP has been confirmed as a crucial factor in DNA binding and phosphorylation, which promote chromosome segregation and cell mitosis 27. Knockdown of TPX2 suppresses the invasion and proliferation of hepatocellular carcinoma cells via the deactivation of AKT signaling and suppression of MMP-2 and MMP-9 gene expression 28.

In addition, a series of small-molecule drugs with potential value for the treatment of cutaneous melanoma were identified via the CMap platform and the screened overlapping gene, which provided clues for identifying new targeted anti-tumor drugs for melanoma. The most significant small molecule, menadione (-0.954), which was highly associated with reversing the status of melanoma cells, has not been investigated for its efficacy and safety in melanoma. Meanwhile, the relationship between verteporfin 1,4-chrysenequinone (-0.95) and melanoma remains unknown. We hypothesized that these drugs might hinder the development of melanoma through the genome or transcriptome. Thus, it is absolutely necessary to investigate the value of the suggested small molecules for melanoma treatment, especially considering their ability to completely reverse the gene expression in melanoma. This will contribute to enhancing our understanding of the therapeutic mechanisms of these candidate small-molecule drugs for melanoma from the perspective of DEGs induced by melanoma.

## Conclusion

In our work, we first conducted integrated microarray analysis to uncover eight key genes that enhance our understanding of the molecular pathogenesis of melanoma initiation and progression. Our results revealed several novel biomarkers and biological pathways that participate in the pathogenesis of cutaneous melanoma, and demonstrated the diagnostic and prognostic value of CDC45, CENPF, DTL, FANCI, GINS2, HJURP, TPX2 and TRIP13. In addition, the identified candidate small-molecule drugs will contribute to the development of novel gene anti-tumor drugs for melanoma. Taken together, we uncovered several promising novel biomarkers in melanoma and provided new insights into about cutaneous melanoma.

## Funding

This study was supported by the Youth Innovation Fund of The First Affiliated Hospital of Zhengzhou University (Grant No. YNQN 2017053) and the Key R&D and Promotion Program of Henan Science and Technology Department (Grant No. 192102310111, 192102310118).

## Figures and Tables

**Table 1:** Clinical properties of cutaneous melanoma patients

	GEO ID	Platform	No. of samples	Death event	Ages (years)	Gender (male/ female)	Primary/Matastatic	adjuvant chemotherapy	Stage (I/II/III/IV)
Melanoma Samples	GSE3189	GPL96	45	39	66.63± 15.78	21/24	12/33	45	2/7/14/22
	GSE7553	GPL570	54	47	72.03 ± 26.37	35/19	6/48	54	0/2/14/38
Normal Samples	GSE3189	GPL96	7	NA	58.68 ± 17.49	3/4	NA	NA	NA
	GSE7553	GPL570	4	NA	62.35 ± 16.50	2/2	NA	NA	NA

NA: Not Applicable

**Table 2:** Functional and pathway enrichment analysis of the overlap DEGs

Category	Term	P value
GOTERM_BP_FAT	GO:0008544~epidermis development	2.67E-20
GOTERM_BP_FAT	GO:0007398~ectoderm development	5.36E-20
GOTERM_BP_FAT	GO:0009913~epidermal cell differentiation	3.80E-10
GOTERM_BP_FAT	GO:0030855~epithelial cell differentiation	1.06E-09
GOTERM_BP_FAT	GO:0030216~keratinocyte differentiation	9.21E-09
GOTERM_CC_FAT	GO:0005911~cell-cell junction	5.65E-12
GOTERM_CC_FAT	GO:0043296~apical junction complex	4.18E-11
GOTERM_CC_FAT	GO:0016327~apicolateral plasma membrane	7.23E-11
GOTERM_CC_FAT	GO:0044459~plasma membrane part	1.64E-10
GOTERM_CC_FAT	GO:0030057~desmosome	3.99E-08
GOTERM_MF_FAT	GO:0004714~transmembrane receptor protein tyrosine kinase activity	2.35E-05
GOTERM_MF_FAT	GO:0005198~structural molecule activity	1.14E-04
GOTERM_MF_FAT	GO:0005200~structural constituent of cytoskeleton	3.01E-04
GOTERM_MF_FAT	GO:0004866~endopeptidase inhibitor activity	0.001161
GOTERM_MF_FAT	GO:0030414~peptidase inhibitor activity	0.001841
KEGG_PATHWAY	hsa04512:ECM-receptor interaction	0.003447
KEGG_PATHWAY	hsa03320:PPAR signaling pathway	0.003522
KEGG_PATHWAY	hsa05200:Pathways in cancer	0.0041
KEGG_PATHWAY	hsa00980:Metabolism of xenobiotics by cytochrome P450	0.006031
KEGG_PATHWAY	hsa00140:Steroid hormone biosynthesis	0.006279

**Table 3:** List of the 20 most significant small molecule drugs that can reverse the tumoral status of melanoma.

Cmap name	Enrichment score	P value
menadione	-0.954	0.00453
1,4-chrysenequinone	-0.95	0.00545
MS-275	-0.938	0.00799
piperlongumine	-0.856	0.04151
difenidol	-0.845	0.00747
Y-27632	-0.835	0.05432
imipenem	-0.814	0.00219
quinostatin	-0.8	0.07859
etomidate	-0.784	0.02063
bezafibrate	-0.76	0.00684
flunarizine	-0.751	0.00774
omeprazole	-0.748	0.00808
camptothecin	-0.747	0.03295
5248896	-0.745	0.12913
latamoxef	-0.743	0.03463
alsterpaullone	-0.74	0.03591
medrysone	-0.738	0.00066
apigenin	-0.735	0.00985
DL-thiorphan	-0.735	0.13957
dequalinium chloride	-0.729	0.01104
